# Efficacy of a one-week course of topical bromfenac combined with corticosteroid for prevention of pseudophakic cystoid macular edema (PCME)

**DOI:** 10.1371/journal.pone.0356992

**Published:** 2026-08-27

**Authors:** Yoon Soo Kim, Myung-Sun Song, Young Sub Eom, Jong Suk Song, Dong Hyun Kim

**Affiliations:** Department of Ophthalmology, Korea University College of Medicine, Seoul, Republic of Korea; University of Warmia, POLAND

## Abstract

**Purpose:**

To compare the efficacy of a 1-week postoperative regimen of topical bromfenac combined with corticosteroid versus corticosteroid monotherapy in preventing pseudophakic cystoid macular edema (PCME).

**Methods:**

This retrospective study included 226 eyes of 226 patients who underwent uneventful phacoemulsification. Patients were divided into Group A (n = 158), receiving topical bromfenac 0.1% twice daily (BID) plus prednisolone acetate 1% (PDE) 6 times daily (6/d) or every 2 hours while awake (q2hrs), and Group B (n = 68), receiving PDE alone 6/d or q2hrs for the first postoperative week. Macular thickness was measured by Spectral-domain optical coherence tomography (SD-OCT) preoperatively and at postoperative week 5. PCME was defined as an increase in central subfield macular thickness (CSMT) with foveal cystoid changes. CSMT changes and PCME incidence were compared. A subgroup analysis evaluated the effect of PDE dosing frequency within the combination arm (6/d vs. q2hrs).

**Results:**

Postoperative absolute and relative CSMT changes were significantly smaller in Group A (13.3 ± 13.0 μm, 5.6 ± 5.3%) than in Group B (27.0 ± 33.7 μm, 11.2 ± 14.3%; *p* = 0.002). The incidence of OCT-defined PCME was lower in Group A than in Group B (5.1% vs. 16.2%; OR = 0.28; 95% CI 0.11–0.72; *p* = 0.006). Among eyes developing PCME, the median CSMT increase was lower in Group A (38.5 μm) than in Group B (80.0 μm; *p* = 0.031). PCME incidence was numerically lower in [Bromfenac BID + PDE q2hrs] subgroup than [Bromfenac BID + PDE 6/d] subgroup (2.0% vs. 10.0%, OR = 0.19; 95% CI 0.04–0.79; *p* = 0.054).

**Conclusion:**

A one-week course of topical bromfenac combined with topical corticosteroid therapy was associated with smaller postoperative increases in macular thickness and a lower incidence of OCT-defined PCME than corticosteroid monotherapy after cataract surgery.

## Introduction

Pseudophakic cystoid macular edema (PCME), also known as Irvine-Gass syndrome, was first described by Irvine in 1953, and later confirmed through fluorescein angiography in 1966 by Gass and Norton [1,2]. Advances in modern small-incision phacoemulsification have lowered incidence of PCME [3], yet a recent systematic review estimated a pooled cumulative incidence of approximately 5% in otherwise uncomplicated cases [4]. PCME remains among the most common causes of suboptimal visual recovery following uneventful cataract surgery [5,6]. The pathogenesis is multifactorial; a principal mechanism involves synthesis of prostaglandins and other pro-inflammatory mediators in the anterior segment due to surgical manipulation, disrupting the blood-aqueous barrier and blood-retinal barrier, leading to increased vascular permeability [3,7–9]. Fluid leakage from perifoveal capillaries results in cystoid spaces—typically within the inner nuclear and outer plexiform layers—and macular thickening [7,10].

Clinically significant PCME may present with blurred vision, metamorphopsia, central scotoma, or reduced contrast or color sensitivity [11]; it has been defined as macular edema accompanied by postoperative best-corrected visual acuity (BCVA) worse than 20/40 in several studies [7]. By contrast, many cases are often asymptomatic and detectable only with fluorescein angiography or optical coherence tomography (OCT) [12–14]. High-resolution spectral-domain OCT (SD-OCT) enables noninvasive, quantitative, and repeatable assessment of retinal thickness, facilitating early detection of subtle cystoid changes [15]. The interval between postoperative weeks 4 and 6 is the peak period for PCME onset, supporting close OCT monitoring during this timeframe [13]. Although most cases of acute PCME resolve spontaneously [7], incomplete resolution can result in permanent visual deterioration, underscoring the need for prevention [6,16]. High-risk eyes—those with intraoperative anterior segment trauma (e.g., iris injury, posterior capsular rupture), preexisting inflammatory conditions (e.g., diabetic retinopathy, uveitis), or macular pathology (e.g., epiretinal membrane)—warrant more aggressive prophylactic strategies [4,17–19].

Perioperative topical corticosteroids and nonsteroidal anti-inflammatory drugs (NSAIDs) remain the foundation of PCME prophylaxis and may act additively or synergistically [5,20–23]. Corticosteroids inhibit phospholipase A2, thereby limiting arachidonic acid release and downstream inflammatory cascades, while NSAIDs suppress prostaglandin synthesis by blocking cyclooxygenase 1 (COX-1) and 2 (COX-2) [24]. Randomized trials and meta-analyses have shown that topical NSAIDs—alone or in combination with corticosteroids—are superior to corticosteroid monotherapy for preventing PCME [25–29]. Most previous combination regimens maintained topical NSAIDs for 4 weeks or longer postoperatively while tapering corticosteroids over a similar period. Nevertheless, optimal dosing schedule and treatment duration remain uncertain [20], and no topical NSAID has an FDA-approved indication specifically for the prevention of PCME. Bromfenac sodium—an FDA-approved topical NSAID for the treatment of postoperative inflammation and ocular pain after cataract surgery—has increased lipophilicity and enhanced ocular penetration, resulting in greater COX-inhibitory potency relative to other NSAIDs [28,30,31]. In the ESCRS PREMED multicenter randomized trial (report 1), a 2-weeks course of topical bromfenac 0.09% combined with dexamethasone 0.1% reduced the incidence of clinically significant PCME compared with either agent alone in nondiabetic patients [32].

Although topical NSAIDs are effective for PCME prophylaxis, postoperative use has been associated with ocular-surface adverse events [33]. In susceptible patients—particularly those with underlying ocular surface disease (OSD)—serious ocular surface complications (e.g., corneal ulceration, stromal melt or perforation) have been reported, albeit infrequently [34–37]. Given the high prevalence of OSD among elderly patients undergoing cataract surgery, prudent precautions are warranted [38]. Dry eye disease (DED), a common phenotype of OSD, often develops or worsens after cataract surgery through multiple mechanisms [39,40]. Postoperative topical NSAIDs have been implicated as a risk factor for aggravation of DED; reduction in conjunctival goblet cell density has been demonstrated [41,42]. Optimal outcomes after cataract surgery also require managing coexisting DED with various topical medications. However, numerous and complex postoperative eye drop regimens often lead to compromised patient compliance. Therefore, the short-term use of topical NSAIDs may not only be beneficial in preventing PCME and postoperative DED aggravation but could also enhance patient adherence.

To address the uncertainty regarding optimal dosage and the practical constraints of safety and adherence, we sought to determine whether a short-course combination regimen could preserve efficacy for prevention of PCME while minimizing NSAID exposure. We conducted a retrospective, comparative evaluation of two perioperative strategies used in routine clinical practice: a one-week course of topical bromfenac sodium 0.1% twice daily combined with topical corticosteroid versus topical corticosteroid monotherapy. The primary endpoints were (i) postoperative change in CSMT measured by SD-OCT and (ii) incidence of OCT-defined PCME.

## Methods

This study was approved by the Institutional Review Board (IRB) of Korea University Anam Hospital (IRB No. 2025AN0346). The study adhered to the tenets of the Declaration of Helsinki. Informed consent was waived with the approval of the IRB of the Korea University Anam Hospital due to the retrospective nature of the study.

We retrospectively reviewed the medical records of 631 eyes from 431 consecutive patients who underwent phacoemulsification cataract surgery at Korea University Anam Hospital between 1 December 2022 and 26 December 2024. Data were accessed for research purposes from 30 July 2025–23 December 2025. Inclusion criteria were as follows: (1) adults who underwent phacoemulsification with posterior chamber intraocular lens implantation; (2) availability of both preoperative and postoperative SD-OCT images. Exclusion criteria were as follows: (1) intraoperative complications associated with elevated PCME risk (e.g., posterior capsular rupture, zonular dialysis, or iris injury); (2) previous intraocular surgery (e.g., pars plana vitrectomy); (3) prior PCME in the fellow eye; (4) the following retinal diseases identified on preoperative examination: cystoid macular edema, choroidal neovascularization, foveoschisis, vitreomacular traction syndrome, or retinal vein/artery occlusion; (5) diagnosed pseudoexfoliation syndrome, Fuchs’ endothelial dystrophy, Sjögren's syndrome, progressive glaucoma, or congenital ocular anomalies; (6) history of intraocular inflammation (e.g., uveitis or endophthalmitis); (7) history of intravitreal anti–vascular endothelial growth factor injection; (8) use of topical prostaglandin analogs, topical corticosteroids, systemic glucocorticoids, or topical NSAIDs within two weeks before surgery; (9) continuation of topical bromfenac or prednisolone acetate beyond one week postoperatively. Patients with diabetes mellitus (DM) or hypertension (HTN) and eyes with epiretinal membrane (ERM) were not excluded. Only one eye per patient was included; if both eyes were eligible, one eye was randomly selected using a computer-generated sequence, stratified by laterality (right or left) and treatment group to maintain an approximately 1:1 laterality distribution.

A total of 226 eyes from 226 patients were included in the analysis. All patients received topical bromfenac sodium 0.1% (Bronuck®; Taejoon Pharm, Seoul, Korea) twice daily (BID) for three days preoperatively. Postoperative anti-inflammatory therapy during the first week defined treatment groups: Group A (Bromfenac Combination Therapy, n = 158) received bromfenac sodium 0.1% BID plus topical prednisolone acetate 1% (Predbell®; Chong Kun Dang Pharm, Seoul, Korea) either six times daily (6/d) or every 2 hours while awake (q2hrs); Group B (Corticosteroid Monotherapy, n = 68) received topical prednisolone acetate 1% alone either 6/d or q2hrs. The postoperative anti-inflammatory regimen reflected a temporal change in routine clinical practice during the study period and was not selected according to cataract grade, operative complexity, or intraoperative findings. From postoperative week 2, all patients were switched to loteprednol etabonate 0.5% (Lotemax®; Bausch & Lomb, Inc., US) four times daily (QID) for four weeks. Antibiotic prophylaxis consisted of topical moxifloxacin 0.5% (Vigamox®; Alcon Laboratories, Inc., US) 6/d or q2hrs for the first week, followed by levofloxacin 1.5% (Cravit®; Santen Pharmaceutical, Japan) QID for the subsequent four weeks. Baseline variables—including age, sex, comorbidities, and cataract grade—were compared between groups. Cataract severity was graded with the Lens Opacities Classification System III (LOCS III).

Macular thickness was measured on SD-OCT (DRI OCT Triton; Topcon, Tokyo, Japan) using the Early Treatment Diabetic Retinopathy Study (ETDRS) retinal thickness map. Mean thickness within the central 1-mm circular region, the concentric 1- to 3-mm annular region, and the concentric 3- to 6-mm annular region were defined as CSMT, parafoveal thickness, and perifoveal thickness, respectively. Changes in macular thickness were calculated by comparing postoperative week 5 (± 7 days) values with baseline. Outcomes were expressed as ‘absolute change’ (μm; postoperative value minus baseline value) and ‘relative change’ (%; absolute change divided by baseline value × 100). Between-group comparisons were performed for baseline values, postoperative values, and absolute/relative changes across the three regions. PCME was defined, in accordance with the PREMED trial, as a ≥ 10% increase in CSMT accompanied by foveal cystoid changes on OCT. Incidence of PCME was compared between groups in all eyes, and within subsets classified according to the presence or absence of known risk factors (DM and ERM). In addition, among eyes that developed PCME, absolute and relative changes in CSMT were compared between treatment groups.

Subgroup analyses were performed according to the dosing frequency of prednisolone acetate 1% (PDE) within the eyes received combination regimen: [Bromfenac BID + PDE q2hrs] (n = 98); [Bromfenac BID + PDE 6/d] (n = 60). Comparative analyses of macular thickness changes and PCME incidence were conducted between these subgroups.

Statistical analyses were performed using GraphPad Prism version 9.4.0 (GraphPad Software, San Diego, CA, USA). The normality of continuous variables was assessed using the Shapiro–Wilk test and visual inspection of histograms and Q–Q plots. Continuous variables were primarily compared using two-sided Welch’s t-test, and sensitivity analyses using the Mann–Whitney U test were performed for variables showing substantial deviation from normality. Categorical variables were compared using the chi-square test or Fisher’s exact test, as appropriate. Odds ratios and 95% confidence intervals for PCME incidence were calculated using the Baptista–Pike (mid-P) method. Absolute and relative changes in CSMT among eyes with PCME were compared using the Mann-Whitney U test. Two-sided *p* values < 0.05 were considered statistically significant. Where applicable, effect estimates are presented with 95% confidence intervals.

## Results

### Baseline characteristics

There were no significant differences in age, sex, or comorbidities such as DM and ERM between Group A and B (Table 1). Nuclear opalescence grade (NO) was significantly higher in Group A than Group B (4.1 ± 0.8 vs. 3.8 ± 0.8; *p* = 0.006). The prevalence of mature nuclear cataract (NO ≥ 5) was 29.8% in Group A and 20.6% in Group B (*p* = 0.155). Cortical (C) and posterior subcapsular (PSC) grades were comparable between groups.

**Table 1:** Baseline characteristics of the study population.

	Bromfenac Combination Therapy (Group A, n = 158)	Corticosteroid Monotherapy (Group B, n = 68)	*P* value
Age (years) [Mean ± SD]	70.2 ± 8.0	69.3 ± 8.1	0.455^*^
Sex, male [n (%)]	62/158 (39.2)	27/68 (39.7)	0.948^†^
DM [n (%)]	43/158 (27.2)	17/68 (25.0)	0.729^†^
ERM [n (%)]	16/158 (10.1)	5/68 (7.4)	0.510^†^
NO ≥ 5 [n (%)]	47/158 (29.8)	14/68 (20.6)	0.155^†^
NO [Mean ± SD]	4.1 ± 0.8	3.8 ± 0.8	**0.006** ^*^
C [Mean ± SD]	3.1 ± 0.5	3.1 ± 0.5	0.692^*^
PSC [Mean ± SD]	1.6 ± 0.8	1.6 ± 0.9	0.560^*^

Statistically significant results (two-sided, *p* < 0.05) are indicated in bold.

Group comparisons were performed using ^*^Welch’s independent t-test and ^†^Chi-square test.

SD: standard deviation; DM: diabetes mellitus; ERM: epiretinal membrane; NO: nuclear opalescence; C: cortical; PSC: posterior subcapsular.

### Macular thickness

Differences in macular thickness are presented in Table 2. At baseline, central subfield macular, parafoveal, and perifoveal thickness was comparable between the groups (all *p* > 0.10). At postoperative week 5, macular thickness in all regions was significantly lower in Group A compared to Group B (CSMT, *p* = 0.007; parafoveal, *p* = 0.003; perifoveal, *p* = 0.015). Specifically, CSMT increased from 232.6 ± 34.3 μm to 245.9 ± 39.4 μm in Group A, and from 239.4 ± 39.1 μm to 266.4 ± 55.2 μm in Group B. Absolute change was 13.3 ± 13.0 μm in Group A and 27.0 ± 33.7 μm in Group B (*p* = 0.002), while relative change was 5.6 ± 5.3% versus 11.2 ± 14.3% (*p* = 0.002). Compared with the bromfenac combination therapy group, the corticosteroid monotherapy group showed greater absolute and relative increases in CSMT, with between-group mean differences of 13.7 μm (95% CI 5.3–22.1) and 5.6 percentage points (95% CI 2.1–9.2), respectively. In the parafoveal region, macular thickness increased from 296.2 ± 21.6 μm to 311.5 ± 26.0 μm in Group A, and from 300.2 ± 20.2 μm to 324.5 ± 30.3 μm in Group B. Absolute change was 15.2 ± 11.1 μm versus 24.2 ± 18.5 μm (*p* < 0.001); relative change was 5.1 ± 3.6% and 8.0 ± 6.0%, respectively (*p* < 0.001). In the perifoveal region, macular thickness increased from 258.6 ± 14.6 μm to 271.0 ± 18.2 μm in Group A, and from 260.2 ± 14.1 μm to 277.9 ± 19.8 μm in Group B. Absolute change was 12.3 ± 8.7 μm versus 17.8 ± 9.7 μm (*p* < 0.001); relative change was 4.8 ± 3.3% versus 6.8 ± 3.6% (*p* < 0.001).

**Table 2:** Comparison of macular thickness between short-term bromfenac combination therapy and corticosteroid monotherapy groups.

	[Mean ± SD]		
	Bromfenac Combination Therapy (Group A, n = 158)	Corticosteroid Monotherapy (Group B, n = 68)	*P* value
**CSMT**			
Baseline (μm)	232.6 ± 34.3	239.4 ± 39.1	0.218
Postoperative (μm)	245.9 ± 39.4	266.4 ± 55.2	**0.007**
Absolute change (μm)	13.3 ± 13.0	27.0 ± 33.7	**0.002**
Relative change (%)	5.6 ± 5.3	11.2 ± 14.3	**0.002**
**Parafoveal Thickness**			
Baseline (μm)	296.2 ± 21.6	300.2 ± 20.2	0.182
Postoperative (μm)	311.5 ± 26.0	324.5 ± 30.3	**0.003**
Absolute change (μm)	15.2 ± 11.1	24.2 ± 18.5	**< 0.001**
Relative change (%)	5.1 ± 3.6	8.0 ± 6.0	**< 0.001**
**Perifoveal Thickness**			
Baseline (μm)	258.6 ± 14.6	260.2 ± 14.1	0.454
Postoperative (μm)	271.0 ± 18.2	277.9 ± 19.8	**0.015**
Absolute change (μm)	12.3 ± 8.7	17.8 ± 9.7	**< 0.001**
Relative change (%)	4.8 ± 3.3	6.8 ± 3.6	**< 0.001**

Statistically significant results (two-sided, *p* < 0.05) are indicated in bold.

Group comparisons were performed using Welch’s independent t-test.

SD: standard deviation; CSMT: central subfield macular thickness.

### Incidence of PCME

The incidence of PCME is summarized in Table 3. PCME occurred in 8 of 158 eyes (5.1%) in Group A and 11 of 68 eyes (16.2%) in Group B, with a significantly lower incidence in Group A (OR = 0.28; 95% CI 0.11–0.72; *p* = 0.006). In prespecified subsets, PCME incidence was lower in Group A than Group B among patients without DM [DM (-)] (5.2% vs 15.7%; OR = 0.30; 95% CI 0.09–0.95; *p* = 0.035), among ERM-negative eyes [ERM (-)] (4.3% vs 14.3%; OR = 0.27; 95% CI 0.09–0.76; *p* = 0.018), and among eyes without either risk factor [DM (–) and ERM (–)] (4.7% vs 14.6%; OR = 0.29; 95% CI 0.10–0.90; *p* = 0.049). Among eyes with any risk factor [DM (+) or ERM (+)], incidence was 5.9% in Group A and 20.0% in Group B, and the difference was not significant (OR = 0.25; 95% CI 0.06–1.04; *p* = 0.092).

**Table 3:** Comparison of PCME incidence between short-term bromfenac combination therapy and corticosteroid monotherapy groups.

	[n (%)]			
	Bromfenac Combination Therapy (Group A, n = 158)	Corticosteroid Monotherapy (Group B, n = 68)	OR (95% CI)	*P* value
All patients	8/158 (5.1%)	11/68 (16.2%)	0.28 (0.11, 0.72)	**0.006** ^*^
DM (-)	6/115 (5.2%)	8/51 (15.7%)	0.30 (0.09, 0.95)	**0.035** ^**†**^
ERM (-)	6/141 (4.3%)	9/63 (14.3%)	0.27 (0.09, 0.76)	**0.018** ^**†**^
DM (-) and ERM (-)	5/107 (4.7%)	7/48 (14.6%)	0.29 (0.10, 0.90)	**0.049** ^**†**^
DM (+) or ERM (+)	3/51 (5.9%)	4/20 (20.0%)	0.25 (0.06, 1.04)	0.092^†^

PCME: pseudophakic cystoid macular edema; OR: odds ratio; CI: confidence interval; DM: diabetes mellitus; ERM: epiretinal membrane.

Group comparisons were performed using ^*^Chi‑square test and ^†^Fisher’s exact test.

Odds ratios and 95% confidence intervals were calculated using the Baptista–Pike method.

Statistically significant results (two-sided, *p* < 0.05) are indicated in bold.

**Fig 1:**
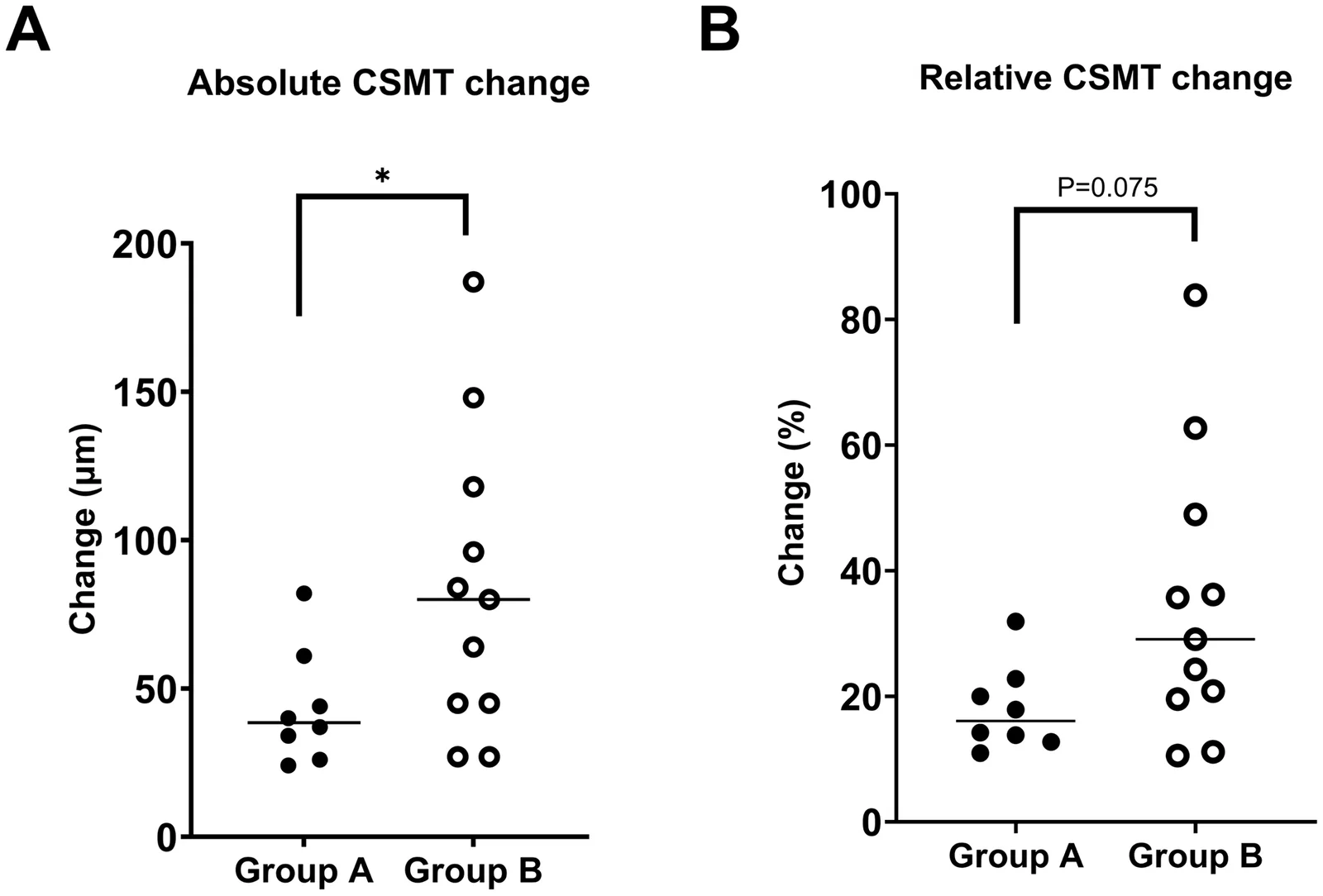
Comparison of absolute and relative changes in CSMT among eyes with PCME.

Fig 1 shows individual-level changes in CSMT among eyes with PCME. Absolute and relative CSMT changes are plotted as dots; black dots denote Group A and white dots denote Group B. The median absolute CSMT increase was significantly smaller in Group A than in Group B (38.5 μm vs. 80.0 μm; *p* = 0.031). The median relative increase was also smaller in Group A (16.1% vs. 29.1%), although this difference was marginally significant (*p* = 0.075). Only one eye in Group B, with severe non-proliferative diabetic retinopathy, required referral to a retina specialist for recurrent PCME, whereas the remaining affected eyes showed complete resolution after adjunctive topical bromfenac administered twice daily for 1–2 months. The case-level clinical characteristics, OCT findings, and subsequent treatment courses of eyes with OCT-defined PCME are summarized in S1 Table.

Absolute and relative changes in central subfield macular thickness (CSMT) in eyes with pseudophakic cystoid macular edema (PCME) are shown as individual dots. Each dot represents an individual eye; black dots indicate Group A, and white dots indicate Group B.

### Subgroup analysis

Baseline characteristics were similar between the subgroups, except that age and NO were higher in [Bromfenac BID + PDE q2hrs] subgroup (Table 4). Baseline macular thickness did not differ between the subgroups, and absolute/relative changes from baseline were also comparable. OCT-defined PCME occurred in 2 of 98 eyes (2.0%) in the [Bromfenac BID + PDE q2hrs] subgroup and in 6 of 60 eyes (10.0%) in the [Bromfenac BID + PDE 6/d] subgroup. Although the odd was numerically lower in [Bromfenac BID + PDE q2hrs] subgroup (OR = 0.19; 95% CI 0.04–0.79), the difference did not reach conventional statistical significance by Fisher’s exact test (*p* = 0.054).

**Table 4:** Comparison of PCME incidence and macular thickness changes between short-term bromfenac combination therapy subgroups (Bromfenac BID + PDE q2hrs vs. Bromfenac BID + PDE 6/d).

	Bromfenac BID + PDE q2hrs (n = 98)	Bromfenac BID + PDE 6/d (n = 60)	*P* value
Age [Mean ± SD]	71.22 ± 8.25	68.42 ± 7.33	**0.028** ^*^
Sex [n (%)]	42/98 (42.9%)	20/60 (33.3%)	0.234^†^
DM [n (%)]	27/98 (27.6%)	16/60 (26.7%)	0.904^†^
ERM [n (%)]	10/98 (10.2%)	6/60 (10.0%)	0.967^†^
NO [Mean ± SD]	4.4 ± 0.8	3.7 ± 0.6	**0.010** ^*^
**CSMT** [Mean ± SD]			
Baseline (μm)	231.8 ± 37.2	233.9 ± 29.24	0.692^*^
Postoperative (μm)	245.0 ± 42.9	247.3 ± 33.1	0.702^*^
Absolute change (μm)	13.2 ± 13.4	13.4 ± 12.4	0.919^*^
Relative change (%)	5.6 ± 5.4	5.7 ± 5.1	0.848^*^
**Parafoveal Thickness** [Mean ± SD]			
Baseline (μm)	295.9 ± 23.5	296.7 ± 18.3	0.820^*^
Postoperative (μm)	310.6 ± 28.2	312.8 ± 22.0	0.581^*^
Absolute change (μm)	14.7 ± 11.6	16.1 ± 10.1	0.422^*^
Relative change (%)	4.9 ± 3.8	5.4 ± 3.3	0.391^*^
**Perifoveal Thickness** [Mean ± SD]			
Baseline (μm)	259.3 ± 15.4	257.5 ± 13.4	0.439^*^
Postoperative (μm)	271.2 ± 19.4	270.5 ± 16.2	0.800^*^
Absolute change (μm)	11.9 ± 9.1	13.0 ± 8.0	0.465^*^
Relative change (%)	4.6 ± 3.5	5.0 ± 3.1	0.390^*^
**PCME incidence** [n (%)]	**2/98 (2.0%)**	**6/60 (10.0%)**	**0.054^**

Statistically significant results (two-sided, *p* < 0.05) are indicated in bold.

Group comparisons were performed using ^*^Welch’s independent t-test, ^†^Chi‑square test and ^Fisher’s exact test. Odds ratios and 95% confidence intervals were calculated using the Baptista–Pike method.

PCME: pseudophakic cystoid macular edema; PDE: prednisolone acetate; bid: twice daily; 6/d: 6 times daily; q2hrs: every 2 hours while awake; OR: odds ratio; CI: confidence interval; SD: standard deviation; DM: diabetes mellitus; ERM: epiretinal membrane; NO: nuclear opalescence; CSMT: central subfield macular thickness.

## Discussion

In this retrospective cohort study, a short-term, 1-week postoperative regimen of bromfenac sodium 0.1% twice daily combined with prednisolone acetate 1% was associated with smaller postoperative macular thickening and a lower incidence of OCT-defined PCME compared with prednisolone acetate monotherapy. OCT-defined PCME occurred in 8 of 158 eyes (5.1%) in the combination regimen group and in 11 of 68 eyes (16.2%) in the corticosteroid-only group, corresponding to a significantly lower incidence in the combination therapy group (OR = 0.28). The findings in the present study align with randomized trials reporting superiority of NSAID-steroid combinations over steroid monotherapy, although most prior trials used 2–4 weeks of NSAID therapy under controlled conditions [25–29,32]. The present study adds to current evidence by suggesting that a short, one-week anti-inflammatory regimen may be associated with favorable anatomical outcomes in routine practice.

The rationale for discontinuing bromfenac after one week was based on practical and biologically plausible considerations. First, the one-week regimen aligned with the routine postoperative 1-week follow-up visit, at which postoperative status was reassessed before transition to the subsequent common anti-inflammatory regimen. Second, because postoperative inflammation is most prominent during the early postoperative period, peaking on postoperative day 1 and then rapidly subsiding, an intensive anti-inflammatory regimen administered during the first postoperative week may be a plausible strategy for PCME prevention [43,44]. Because the present study did not directly compare one-week therapy with longer NSAID regimens, it cannot determine whether one week is the optimal duration of bromfenac prophylaxis. Nevertheless, despite the relatively advanced cataract severity in the study cohort, the incidence of OCT-defined PCME observed in this study was broadly consistent with rates reported in previous studies [25–29]. Moreover, the pharmacologic characteristics of bromfenac—specifically its rapid corneal penetration, sustained intraocular concentrations, and greater COX inhibitory potency compared with other topical NSAIDs—support the biologic plausibility of short-course bromfenac use during the period of highest inflammatory activity [28,31,45]. In the exploratory subgroup analysis, despite slightly higher baseline age and nuclear opacity, the [Bromfenac BID + PDE q2hrs] group showed a numerically lower PCME incidence (2.0%) compared with the [Bromfenac BID + PDE 6/d] group (10.0%). A higher dosing frequency of corticosteroid during the early postoperative period may provide additional preventive benefit. However, this difference did not reach conventional statistical significance and should be interpreted cautiously.

DM and ERM are established risk factors for PCME, with reported relative risks of 2.87 (95% CI 1.96–4.21) and 4.51 (95% CI 3.06–6.64) [4]. A lower incidence of OCT-defined PCME was observed with the one-week bromfenac combination regimen, both in the overall cohort and in subsets of eyes with DM or ERM. Although statistical significance was not achieved in the DM or ERM subgroup, the consistently contrasting incidence rates (5.9% vs. 20.0%) may reflect limited statistical power due to the small number of events.

Following cataract surgery, patients are commonly prescribed DED treating agents, including diquafosol, rebamipide, cyclosporine, and artificial tears, to mitigate postoperative dry eye. Effective management of this condition is a crucial factor in improving visual quality and patient satisfaction [46]. However, the high number of prescribed drops can compromise patient adherence to complex eye-drop regimens. A previous Japanese study, for instance, reported that only 10.2% of participants adhered to the specified instillation frequency in the package insert [47]. A shorter postoperative NSAID regimen may have potential practical advantages by reducing the duration of topical NSAID exposure and simplifying postoperative eye-drop regimens. This may be relevant because patients undergoing cataract surgery are often elderly and may have coexisting ocular surface disease or use multiple postoperative topical medications. However, ocular surface parameters and adherence measures were not evaluated in the present study. Therefore, whether a one-week bromfenac regimen improves ocular surface safety, tolerability, or treatment adherence compared with longer NSAID regimens remains to be determined in future prospective studies.

This study has several limitations. First, its retrospective, non-randomized design is susceptible to selection bias, time-period effects, and residual confounding. Although most measured baseline characteristics were comparable between the groups, nuclear opalescence grade differed significantly. Denser nuclear cataracts may require greater phacoemulsification energy and may thereby increase postoperative inflammatory burden. However, surgical energy and operative complexity parameters, such as cumulative dissipated energy, ultrasound time, surgical duration, and intraoperative floppy iris syndrome, were not consistently available in the retrospective records. In addition, the small number of OCT-defined PCME events precluded robust multivariable adjustment or propensity score analysis; therefore, residual confounding cannot be excluded. Second, postoperative assessment was limited to the early 4–6 week period. Although this timeframe is appropriate for detecting early OCT-defined PCME, it does not allow assessment of the long-term course of PCME, including persistence, late-onset occurrence, recurrence, or long-term visual outcomes. Third, the present study did not compare a one-week regimen with longer NSAID regimens, and direct comparisons with prior studies using longer NSAID regimens are precluded by differences in study design, treatment protocols, study populations, and outcome definitions. Further prospective randomized trials directly comparing different durations of postoperative NSAID therapy are required. Fourth, treatment adherence and safety outcomes were not systematically assessed. Future prospective studies should incorporate adherence measures and ocular surface safety assessments, such as tear breakup time, ocular surface staining, and meibomian gland evaluation, to better define the clinical utility of short-course bromfenac therapy.

In conclusion, in this retrospective cohort, a one-week postoperative regimen of topical bromfenac combined with corticosteroid therapy was associated with smaller increases in macular thickness and a lower incidence of OCT-defined PCME compared with corticosteroid monotherapy.

## Supporting information

S1 Table Clinical and OCT characteristics of eyes with OCT-defined PCME. (DOCX)
