# Supplementary material for: Efficacy of a one-week course of topical bromfenac combined with corticosteroid for prevention of pseudophakic cystoid macular edema (PCME)
Source: PLoS One. 2026 Aug 27;21(8):e0356992. doi: 10.1371/journal.pone.0356992 (PMC13521450; doi:10.1371/journal.pone.0356992)
Supplement: Supplementary file 1 [file pone.0356992.s001.docx]

**S1 Table. Clinical and OCT characteristics of eyes with OCT-defined PCME.**

| **Case No.** | **Treatment group** | **PDE regimen** | **DM** | **ERM** | **NO grade** | **Baseline CSMT, μm** | **Postoperative CSMT, μm** | **Absolute CSMT change, μm** | **Relative CSMT change, %** | **Additional treatment/ course** |
| --- | --- | --- | --- | --- | --- | --- | --- | --- | --- | --- |
| 1 | Group A | 6/d | Y | N | 4 | 219 | 243 | 24 | 11.0 | Adjunctive Bromfenac BID; Resolved |
| 2 | Group A | 6/d | N | N | 4 | 246 | 290 | 44 | 17.9 | Adjunctive Bromfenac BID; Resolved |
| 3 | Group A | 6/d | N | N | 4 | 200 | 240 | 40 | 20.0 | Adjunctive Bromfenac BID; Resolved |
| 4 | Group A | 6/d | N | Y | 3 | 268 | 329 | 61 | 22.8 | Adjunctive Bromfenac BID; Resolved |
| 5 | Group A | 6/d | N | N | 3 | 204 | 230 | 26 | 12.8 | Adjunctive Bromfenac BID; Resolved |
| 6 | Group A | q2hrs | N | N | 4 | 257 | 339 | 82 | 31.9 | Adjunctive Bromfenac BID; Resolved |
| 7 | Group A | q2hrs | N | N | 3 | 246 | 280 | 34 | 13.8 | Adjunctive Bromfenac BID; Lost to follow-up |
| 8 | Group A | 6/d | Y | Y | 3 | 260 | 297 | 37 | 14.2 | Adjunctive Bromfenac BID; Resolved |
| 9 | Group B | 6/d | N | N | 2 | 235 | 319 | 84 | 35.7 | Adjunctive Bromfenac BID; Resolved |
| 10 | Group B | 6/d | N | Y | 5 | 395 | 491 | 96 | 24.3 | Adjunctive Bromfenac BID; Resolved |
| 11 | Group B | 6/d | N | N | 5 | 241 | 359 | 118 | 49.0 | Adjunctive Bromfenac BID; Resolved |
| 12 | Group B | 6/d | N | N | 5 | 221 | 301 | 80 | 36.2 | Adjunctive Bromfenac BID; Resolved |
| 13 | Group B | 6/d | Y | N | 3 | 230 | 275 | 45 | 19.6 | Adjunctive Bromfenac BID; Resolved |
| 14 | Group B | 6/d | N | N | 3 | 236 | 384 | 148 | 62.7 | Adjunctive Bromfenac BID; Resolved |
| 15 | Group B | 6/d | N | N | 3 | 220 | 284 | 64 | 29.1 | Adjunctive Bromfenac BID; Resolved |
| 16 | Group B | 6/d | N | N | 3 | 223 | 410 | 187 | 83.9 | Adjunctive Bromfenac BID; Resolved |
| 17 | Group B | 6/d | Y | Y | 4 | 242 | 269 | 27 | 11.2 | Adjunctive Bromfenac BID; Recurrent PCME requiring additional retinal treatment; Resolved |
| 18 | Group B | 6/d | Y | N | 5 | 216 | 261 | 45 | 20.8 | Adjunctive Bromfenac BID; Resolved |
| 19 | Group B | q2hrs | N | N | 3 | 256 | 283 | 27 | 10.6 | Adjunctive Bromfenac BID; Lost to follow-up |

PCME: pseudophakic cystoid macular edema; OCT: optical coherence tomography; CSMT: central subfield macular thickness; DM: diabetes mellitus; ERM: epiretinal membrane; NO: nuclear opalescence; PDE: prednisolone acetate 1%; 6/d: 6 times daily; q2hrs: every 2 hours while awake; BID: twice daily.

Group A indicates the bromfenac combination therapy group, which received topical bromfenac 0.1% BID combined with topical prednisolone acetate 1% during the first postoperative week; Group B indicates the corticosteroid monotherapy group, which received topical prednisolone acetate 1% alone during the first postoperative week.

Absolute CSMT change was calculated as postoperative CSMT − baseline CSMT.

Relative CSMT change was calculated as [(postoperative CSMT − baseline CSMT) / baseline CSMT] × 100.

Case numbers are anonymized and do not correspond to patient identifiers. Age, sex, and laterality were removed to reduce the risk of indirect identification.
